# Survival Rates and Associated Factors of Colorectal Cancer Patients in Brunei Darussalam

**DOI:** 10.31557/APJCP.2020.21.1.259

**Published:** 2020

**Authors:** Elvynna Leong, Sok King Ong, Fadhliah Madli, Abby Tan, Daphne Lai, Norwani Basir, Noraslinah Ramlee, Vui Heng Chong

**Affiliations:** 1 *Faculty of Science, *; 2 *Institute of Applied Data Analytics, Universiti Brunei Darussalam, Jln Tungku Link, *; 3 *NCD Prevention Unit, Ministry of Health, Commonwealth Drive, *; 4 *Gastroenterology and Hepatology Unit, Raja Isteri Pengiran Anak Saleha Hospital, *; 5 *Early Detection and Cancer Prevention Services, Pantai Jerudong Specialist Centre, Brunei Darussalam. *

**Keywords:** Colorectal cancer, prognostic factor, survival rate, hazard rate, Cox PH, Brunei Darussalam

## Abstract

**Background:**

Colorectal cancer (CRC) is the third most common cancer in both men and women. In most Asian countries, both the incidence and mortality rates of CRC are gradually increasing. In Brunei Darussalam, CRC ranks first and second in lifetime risk among men and women respectively. This study aims to report the overall survival rates and associated factors of CRC in Brunei Darussalam.

**Methods:**

This is a retrospective study examining CRC data for the period 2007 to 2017 retrieved from a population based cancer registry in Brunei Darussalam. A total of 728 patients were included in the analysis. Kaplan Meier method was used to estimate survival rates. Univariate analysis using log-rank test was used to examine the differences in survival between groups. Multivariate analysis using Cox PH regression was used to estimate hazard of death and obtain significant predictors that influence CRC patients’ survival.

**Results:**

The median survival time for colorectal, colon and rectal cancer patients were 57.0, 85.8 and 40.0 months respectively. The overall 1-, 3- and 5- year survival rates for CRC patients were 78.0%, 57.7% and 49.6% respectively. In univariate analysis, age at diagnosis, ethnicity, cancer stage, tumour location and histology were found to have significant difference in CRC patients’ survival. In the Cox PH analysis, older age (≥70 years), cancer stage, ethnicity and other histological type were determined as associated factors of CRC patients’ survival.

**Conclusion:**

This study found the overall 5-year survival rate of CRC in Brunei Darussalam is similar to that in some Asian countries such as Singapore and Malaysia. However, more efforts need to be carried out in order to raise awareness of CRC and improve the survival of CRC patients**.**

## Introduction

CRC is ranked the third most commonly diagnosed malignancy in men and women, and accounted for second most common cancer deaths in 2018 (WHO, 2018). The incidence rate of CRC in 2018 was reported to be significantly higher in men (1,026,215) than women (823,303) (WHO, 2018). The global burden of CRC was estimated to increase by 60% which accounts for more than 2.2 million new cases and 1.1 million deaths by 2030 (Arnold et al., 2017). 

The rising incidence in CRC was suggested to be associated with the improving socioeconomic status and increased westernized lifestyle (Veetil et al., 2016; Center et al., 2009; Goss et al., 2014). Apart from that, other risk factors such as ageing population, uncontrolled use of tobacco and intake of alcohol, and physical inactivity could also contribute to the prevalence of CRC (Goss et al., 2014). Across the Asian countries, both the incidence and mortality rates of CRC are rapidly increasing although in some developed countries particularly Japan, South Korea, Hong Kong and Singapore, the mortality rates have declined and the decline started in the younger age groups (Shin et al., 2013). 

In Brunei Darussalam, CRC incidence rate is also rising in both Bruneian men and women which recorded 1,126 cases from 1986 until 2014 (Koh et al., 2015). From 2011 to 2015, CRC ranks first and second in term of lifetime risk among men (5.0%) and women (4.5%) in Brunei Darussalam (Ong et al., 2018). Brunei Darussalam is a country in South East Asia with an estimated population of 442,400. The Department of Economic Planning and Development (DEPD), Ministry of Finance and Economy (2018) reported that the major ethnic groups are Malay (65.7%), Chinese (10.3%) and other ethnicity (24.0%). Brunei Darussalam is divided into four districts namely Brunei-Muara (69.4%), Tutong (11.6%), Belait (16.5%) and Temburong (2.5%) (DEPD, 2018). This study was conducted to estimate the survival rates and explore the associated factors of CRC patients in Brunei Darussalam.

## Materials and Methods

This study was conducted using de-identified data from the Brunei Darussalam Cancer Registry (BDCR) recorded from 1st January 2007 to 31st December 2017. All CRC patients (local citizen and permanent residents of Brunei Darussalam) registered with the local health services were included in this study. Foreign nationals were excluded from the study. Ethical approval for this study was obtained from PAPRSB Institute of Health Science Research and Ethics Committee (IHSREC) and the Medical and Health Research Ethics committee of Ministry of Health (MHREC), Brunei Darussalam [Ref: UBD/PAPRSBIHSREC/2018/149, dated 21^st^ January 2019].

The data extracted from the medical records included demographic characteristics such as age at diagnosis, gender, ethnicity (Malay, Chinese, Others), district (Brunei-Muara, Tutong, Belait, Temburong), and clinical characteristics such as cancer stage (Localized, Regional, Distant), tumour location (Right colon, Left colon, Overlapping and Colon Not Otherwise Specified (NOS), Rectum) and histology (Adenocarcinoma (AC), Mucinous Adenocarcinoma (MAC) and Others). Cancer stages were categorized using a staging system developed by Surveillance, Epidemiology and End Results program (SEER) which classifies CRC cancer cases into localized (SEER Stage 1 equivalent to TNM Stage I and IIa: T1-T3/N0/M0), regional (SEER Stage 2-5 equivalent to TNM Stage IIa and IIb and III: T3-T4/AnyN/Any T/N1,2/M0), and distant metastasis (SEER Stage 7 equivalent to TNM Stage IV: AnyT/AnyM/M1) (Young et al., 2012; Cunningham et al., 2008). Tumour location and histology were coded according to the International Classification of Diseases for Oncology, third edition, topographical codes (ICD-0-3) (Fritz et al., 2000). Histology was categorized as adenocarcinoma, AC (ICD-0-3, codes 8140, 8145, 8210, 8260, 8263, 8570, 8574), mucinous adenocarcinoma, MAC (8480, 8481) and others (8000, 8010, 8246, 8070, 8013, 8800, 8850, 8936, 8240, 8490). Location of tumour was categorized into right colon (cecum, ascending colon, hepatic flexure and transverse colon; C180 and C182-C184), left colon (splenic flexure, descending colon and sigmoid colon; C185-C187), overlapping and colon not otherwise specified, NOS (C188-C189), and rectum (C20). Cancer cases of appendix (C181) were excluded from the study.

The analyses were performed using R statistical software. Survival time was defined as the period of time from diagnosis to death or end of follow-up. Patients who are still alive or lost to follow-up at the end of the study period were right censored. Overall survival rates were calculated using the Kaplan-Meier method. Overall survival was defined as the period of time from diagnosis to death or end of follow-up, due to any cause. Univariate analysis, using the log-rank test, was used to examine the differences in survival between groups. The median survival time was estimated at which the survival probability is 0.5. Multivariate analysis, using the Cox Proportional Hazard (PH) regression model, was used to estimate the hazard ratios (HR) of associated factors on survival and to select the significant predictors for CRC patients’ survival. We examined the proportional hazard assumption and measured the goodness of fit for model adequacy. For all analyses, the level of statistical significance was set at 5%. Confidence intervals of 95% were reported where appropriate.

## Results

A total of 728 patients were included in the analysis. The mean age at diagnosis for CRC patients was 60.5 years (standard deviation = 14.4), with a majority of them in the age group of 50-59 years (27.9%), shown in Table 1. Malay was the most ethnic group diagnosed with CRC (73.2%), followed by Chinese (20.9%) and other ethnicity (5.9%). Majority of the CRC patients resided in the Brunei Muara district (65.4%) followed by Belait (18.4%), Tutong (13.7%) and Temburong (2.5%). A total of 56.3% CRC patients were men while the remaining were women (43.7%). Most CRC patients had histological type AC (83.1%). Majority of the patients were diagnosed at regional stage (41.5%), followed by distant metastasis (30.4%) and localized stage (28.1%). 

A total of 457 (62.8%) cases were diagnosed with colon cancer whereas the remaining 271 (37.2%) cases were diagnosed with rectum cancer. Majority of the colon cancer patients were 70 years and above (30.0%) followed by the age group 50-59 years (27.8%) while most rectum cancer cases were in the age group of 50-59 years (28.0%) followed by age 70 years and above (25.1%), presented in Table 1. Slightly more than half of the colon cancer patients were male (53.4%). There were also more male rectum cancer patients than women (61.3% vs 38.7%). Malay ethnicity group recorded the highest number of colon cancer cases (72.6%) followed by Chinese ethnicity (21.9%). Similar pattern can be seen for rectum cancer patients. 

**Figure 1:**
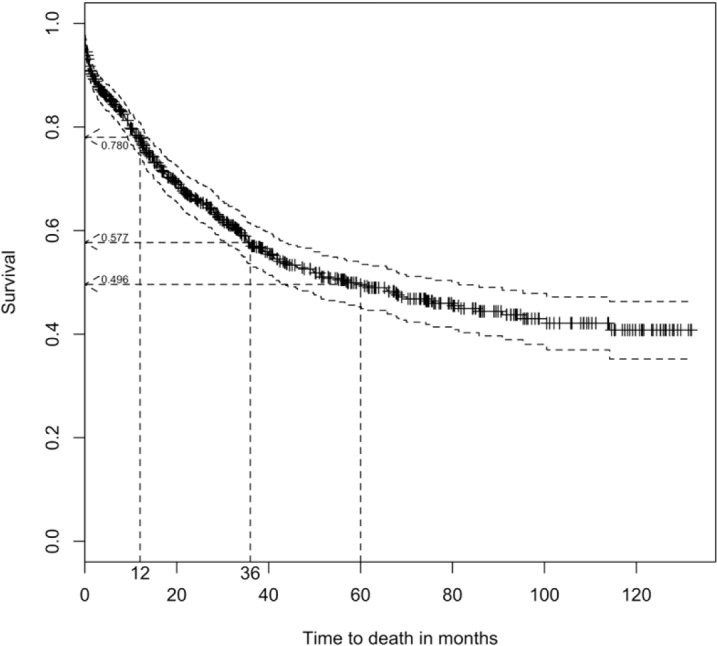
Kaplan Meier Overall Survival Estimates among CRC Patients with 95% Confidence Interval

**Figure 2:**
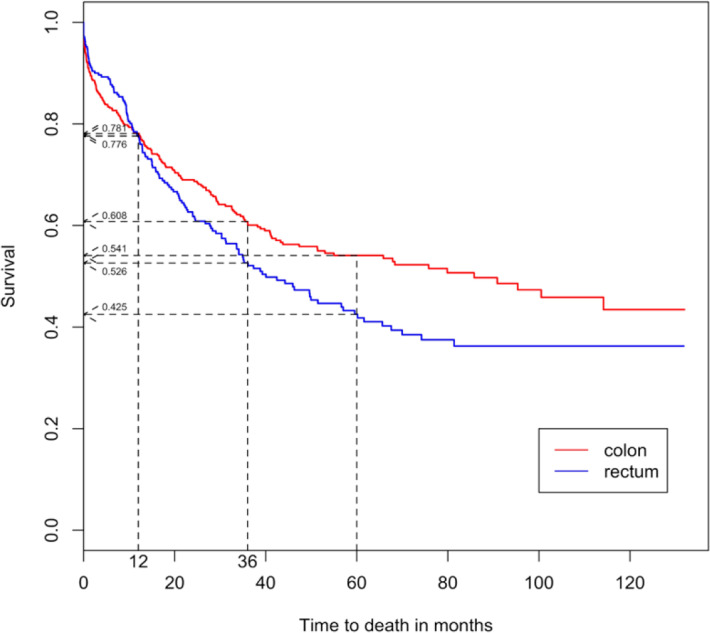
Survival Curves of Colon and Rectum Cancer Patients

The overall survival rates at 1, 3 and 5 years for CRC patients in Brunei Darussalam were 78.0%, 57.7% and 49.6% respectively (Figure 1) and the median survival was 57.0 (95% C.I.: 42.4-79.9) months, shown in Table 2. The 1, 3 and 5-year survival rates of colon cancer were 78.1%, 60.8% and 54.1% respectively while for rectum cancer, the survival rates were 77.6%, 52.6% and 42.5% at 1, 3 and 5 years respectively. The survival curves of both colon and rectum cancers were illustrated in Figure 2. The median survival for colon cancer was found to be much higher than rectum cancer (85.8 vs 40.0 months).

Table 1 also summarises the median survival times and survival rates according to the covariates. The 5-year survival rate was highest for age group 40 years and below (64.9%) followed by 50-59 year (52.9%), 60-69 years (51.0%), 40-49 years (49.9%) and 70 years and above (40.9%). CRC patients residing in the Belait district had the highest 5-year survival rate (54.4%) followed by Brunei-Muara (53.9%), Temburong (49.5%) and Tutong (41.6%). The median survival time was highest for patients living in Belait (75.8 months) followed by Brunei Muara (74.2 months), Tutong (38.8 months) and Temburong (31.3 months). However, this study found no significant difference in survival between the four districts (p = 0.6240). Malay ethnic group had the lowest 5-year survival rate of 44.4% followed by Chinese patients and other ethnicity group at 58.5% and 82.7% respectively. 

**Figure 3:**
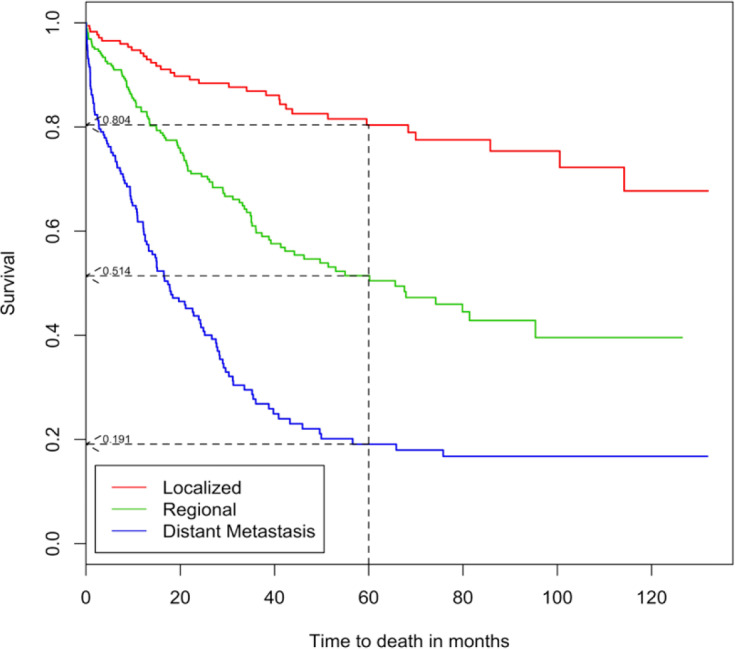
Survival Curves by Cancer Stage of CRC Patients

CRC male patients had lower median survival time of 56.6 months and 5-year survival rate of 48.5% when compared to female patients in which their median survival time and 5-year survival rates were 65.6 months and 51.0% respectively. However, there was no significant difference in survival between gender (p = 0.8940). The 5-year survival rates for cancer stages were 80.4%, 51.4% and 19.1% for localized, regional and distant metastasis respectively (Figure 3). Rectal cancer patients (42.5%) had the worst 5-year survival rate while right-sided colon patients had the best 5-year survival rate of 74.6%, followed by left-colon (54.8%) and overlapping or colon, NOS (49.0%). As for the histological subtype, AC had the highest median survival time and 5-year survival rate (75.8 months and 52.9%) compared to MAC (39.2 months and 45.4%) and other histology (16.2 months and 29.6%). There was a significant difference in CRC patients’ survival between age groups, ethnicity, cancer stages, tumour location and histology based on the log-rank tests (p < 0.05). 

Cox PH regression analyses found that age at diagnosis, cancer stage, ethnicity and histology were the significant associated factors for CRC patients’ survival (p < 0.05), shown in Table 3. No violations of proportional hazard assumptions were observed. For age at diagnosis, the expected hazard of death was 2.54 times higher for a CRC patient in the age group 70 years and above compared to a patient below 40 years [HR = 2.54, 95% C.I.: 1.43 – 4.49, p = 0.0013]. For cancer stage, the hazard of death for patients diagnosed with regional stage was 3.08 times higher as compared to patients diagnosed with localized stage [HR = 3.08, 95% C.I.: 2.08 – 4.56, p < 0.001]. However, for patients diagnosed with distant metastasis, the hazard of death was 7.12 times higher as compared to patients diagnosed with regional stage [HR = 7.12, 95% C.I.: 4.82 – 10.51, p < 0.001]. For ethnicity, the hazard of death for a Chinese CRC patient was 0.62 times lower as compared to a Malay CRC patient [HR = 0.62, 95% C.I = 0.43 - 0.88, p = 0.0073] whereas the expected hazard of a CRC patient with other ethnicity was 0.33 times lower as compared to a Malay CRC patient [HR = 0.33, 95% C.I = 0.14 – 0.74, p = 0.0071]. For histology, the hazard of death of a patient with other morphology was 1.70 times higher as compared to a patient with histological type AC [HR = 1.70, 95% C.I.: 1.18 – 2.45, p = 0.0043]. 

**Table 1:** Distribution of CRC Cases, Median Survival Times and 5-year Survival Rates of CRC Patients by Selected Demographic and Pathological Characteristics

	No. of cases n (%)			Median survival times (months)	5-year SR (%)	Log-rank test
	Total	Colon	Rectum			
Age at diagnosis						p<0.0001*
< 40	57 (7.8)	37 (8.1)	20 (7.4)	-	64.9	
40-49	102 (14.0)	59 (12.9)	43 (15.9)	57.0	49.9	
50-59	203 (27.9)	127 (27.8)	76 (28.0)	67.9	52.9	
60-69	161 (22.1)	97 (21.2)	64 (23.6)	90.8	51.0	
≥ 70	205 (28.2)	137 (30.0)	68 (25.1)	28.4	40.9	
District						p=0.6240
Brunei-Muara	438 (65.4)	269 (64.0)	169 (67.6)	74.2	53.9	
Tutong	92 (13.7)	49 (11.7)	43 (17.2)	38.8	41.6	
Belait	123 (18.4)	90 (21.4)	33 (13.2)	75.8	54.4	
Temburong	17 (2.5)	12 (2.9)	5 (2.0)	31.3	49.5	
Ethnicity						p=0.0008*
Malay	533 (73.2)	332 (72.6)	201 (74.2)	41.1	44.4	
Chinese	152 (20.9)	100 (21.9)	52 (19.2)	100.5	58.5	
Others	43 (5.9)	25 (5.5)	18 (6.6)	-	82.7	
Gender						p=0.8940
Male	410 (56.3)	244 (53.4)	166 (61.3)	56.6	48.5	
Female	318 (43.7)	213 (46.6)	105 (38.7)	65.6	51.0	
Stage						p<0.0001*
Localized	175 (28.1)	111 (28.5)	64 (27.6)	-	80.4	
Regional	258 (41.5)	161 (41.3)	97 (41.8)	65.6	51.4	
Distant-metastasis	189 (30.4)	118 (30.3)	71 (30.6)	17.4	19.1	
Tumour location						p=0.0137*
Right colon	65 (8.9)	65 (14.2)	-	-	74.6	
Left colon	192 (26.4)	192 (42.0)	-	79.9	54.8	
Overlapping or colon, NOS	200 (27.5)	200 (43.8)	-	51.4	49.0	
Rectum	271 (37.2)	-	271 (100.0)	40.0	42.5	
Histology						p < 0.0001*
AC	605 (83.1)	380 (83.2)	225 (83.0)	75.8	52.9	
MAC	32 (4.4)	19 (4.2)	13 (4.8)	39.2	45.4	
Others	91 (12.5)	58 (12.7)	33 (12.2)	16.2	29.6	

AC, Adenocarcinoma; MAC, Mucinous adenocarcinoma; SR, overall survival rate; *Statistically significant (p < 0.05).

**Table 2:** 1-, 3-, and 5- Year Overall Survival Rate and Median Survival for CRC Patients

Cancer location	SR, % (95%C.I.)	Median survival (months) (95%C.I.)
CRC (n = 728)		57.0 (42.4 – 79.9)
1 year	78.0 (74.7 – 80.9)	
3 year	57.7 (53.6 – 61.5)	
5 year	49.6 (45.3 – 53.8)	
Colon cancer (n = 457)		85.8 (51.4 - NA)
1 year	78.1 (74.0 – 81.7)	
3 year	60.8 (55.7 – 65.5)	
5 year	54.1 (48.6 – 59.2)	
Rectum cancer (n = 271)		40.0 (31.3 - 57)
1 year	77.6 (72.0 – 82.3)	
3 year	52.6 (45.9 – 58.9)	
5 year	42.5 (35.6 – 49.3)	

SR, overall survival rate; NA, Not Available.

**Table 3:** Multivariate Analysis (Cox Proportional Hazards Regression Model) of Factors Affecting Overall CRC Survival

Variables	HR	95% C.I.	p-value*
Age at diagnosis			
<40	1.000 (Reference)	-	-
40-49	1.572	0.851 – 2.907	0.1489
50-59	1.498	0.845 – 2.659	0.1669
60-69	1.488	0.822 – 2.693	0.1895
≥ 70	2.538	1.434 – 4.492	0.0013
Stage			
Localized	1.000 (Reference)	-	-
Regional	3.079	2.077 – 4.564	< 0.0001
Distant-metastasis	7.117	4.820 – 10.508	< 0.0001
Ethnicity			
Malay	1.000 (Reference)	-	-
Chinese	0.616	0.433 – 0.878	0.0073
Others	0.325	0.144 – 0.737	0.0071
Histology			
AC	1.000 (Reference)	-	-
MAC	1.240	0.690 – 2.227	0.4729
Others	1.703	1.182 – 2.454	0.0043

HR, Hazard Ratio; C.I., Confidence Interval; *p-value for Wald statistics; AC (Adenocarcinoma); MAC (Mucinous adenocarcinoma).

## Discussion

This is the first study in Brunei Darussalam looking at the overall survival rates and associated factors of CRC patients. The overall 5-year survival rate for CRC patients in Brunei Darussalam was 49.6%, comparable to other Asia countries such as Singapore with 51.0% for male and 52.8% for female (NRDO, 2015) and Malaysia with 48.7% (Hasan et al. 2016). Thailand reported lower overall 5-year survival rate of 36.9% (Phimha et al., 2019) while China has higher overall 5-year survival rate of 68% as reported in Yuan et al., (2013). 

This study also showed a higher proportion of colon cancer patients than rectum cancer patients in Brunei Darussalam, consistent with other studies in which they reported the proportion of rectum cancer patients is gradually decreasing in some developed countries (Ji et al., 1998; Toyoda et al., 2009) and some experts suggested that dietary patterns and lifestyle changes could have contributed to the higher incidence of colon cancer cases (Giovannucci and Willett, 1994; Chiu et al., 2003). This study found the 5-year survival rates for colon cancer and rectum cancer to be 54.1% and 42.5% respectively. Several studies also reported the 5-year survival rate of colon cancer to be higher than that of rectum cancer such as in Malaysia (43.9% and 22.8%) (Ghazali et al., 2010), China (69.0% and 66.0%) (Yuan et al., 2013), and Thailand (41.4% and 31.6%) (Laohavinij et al., 2010).

Patients’ survival is significantly influenced by several prognostic factors. In line with other studies (Laohavinij et al., 2010; Lin et al., 2015), this analysis found that age at diagnosis was a significant associated factor for CRC patients’ survival. Our study showed that older patients (≥70) followed by patients in the age group 40-49 had the worst 5-year survival rate compared to other age groups. Some studies suggested the poorer overall survival in younger patients could be due to the cancer in younger patients is more aggressive and less responsive to treatments (Chou et al., 2011; O’Connell et al., 2004). However, Zhang (2010) reported no difference in survival according to age.

Cancer stage is known to be the most important predictor which affects the CRC patients’ survival (Lin et al., 2015; Magaji et al., 2017; Mehrkhani et al., 2009). This study found there was a significant difference between cancer stage and survival. The 5-year survival rate for CRC in this study was 80.4%, 51.4% and 19.1% for localized, regional and distant stage, respectively. Jordan recorded 5-year survival rate of 72.1%, 53.8% and 22.6% (Sharkas et al., 2017) while Saudi Arabia recorded 5-year survival rate of 63.3%, 50.2% and 14.7% for localized, regional and distant stage, respectively (Al-Ahwal et al., 2013). In our study, the highest hazard of death (HR = 7.1) was found to be in patients diagnosed with distant metastasis followed by regional stage (HR = 3.1) compared to patients diagnosed with localized stage. Compared to our study, Jordan had lower hazard of death for both distant metastasis (HR = 4.5) and regional (HR = 1.8) stages diagnosed patients (Sharkas et al., 2017).

The results from this study found ethnicity to be another predictor for CRC patients’ survival, similar to a study in Malaysia (Magaji et al., 2017). An estimate of 73.2% in this study was from the Malay ethnic group. This study found that the 5-year survival rate for Chinese (58.5%) was higher than the Malays (44.4%). This is consistent with the survival rates in a study in Malaysia, in which the 5-year survival rates for Malay was 46.4% and Chinese was 49.6% (Hasan et al., 2016). In Singapore, the survival of Malay patients was also lower compared to Chinese (Du et al., 2002). Lower survival rates in Malays might be due to social and cultural differences in attitudes toward catastrophic illnesses as Malays are less willing to pursue aggressive therapies (Du et al., 2002).

Univariate and multivariate survival analysis both showed histological type to be an associated factor for CRC patients’ survival in this study. Our analysis showed that patients with histological type AC had significant higher 5-year survival rate compared to MAC (52.9% vs 45.4%), similar to a study with metastatic CRC patients which reported that histological type MAC had poor overall survival and overall response rate to chemotherapy compared to patients with histological type AC (Mekenkamp et al., 2012). However, we found that there was no difference in hazard ratio between histological type MAC and AC while patients diagnosed with other histological type was found to have higher hazard compared to histological type AC (HR = 1.70). This study did not find gender and tumour location to be significant predictors for CRC patients’ survival, consistent with other studies (Hasan et al., 2016; Ghazali et al., 2010; Zhang et al., 2014). However, Magaji (2017) reported male as a prognostic factors for poorer survival rate.

There are some limitations in the study. Other significant predictors for CRC patients’ survival that have been studied include primary tumour size, involvement of lymph nodes, treatment modalities (primary surgery or neoadjuvant chemotherapy) and tumour grade (Hasan et al., 2016; Lin et al., 2015; Magaji et al., 2017). Preoperative levels of serum CEA, whereby presence of ≥ 5.0 ng/ml have an adverse impact on survival that is independent of tumour stage (Harrison et al., 1997; Park et al., 2009; Thirunavukarasu et al., 2011; Thirunavukarasu et al., 2015). Preliminary studies suggest molecular features, such as MMR, BRAF and RAS mutations, may also influence outcome, independent of stage at presentation (Sepulveda et al., 2017). Unfortunately, these factors were not available in the data used in this study. In addition, socioeconomic status and quality of survivorship care, which been linked to cancer patient survival, should be explored in future studies as inadequate accessibility to healthcare could result in delay of detecting cancer at an early stage and receiving necessary care and treatment (Kong, 2010). Another limitation is the presence of missing data, commonly seen in all retrospective studies. Despite the limitations, these results provided a glimpse into the present situation about survival rate of CRC patients in Brunei Darussalam and could serve as a basis for further research, future cancer education and screening programs.

CRC screening has been shown to affect the outcomes of CRC with reduction in incidence and mortality. As CRC takes years to develop, screening allows detection and removal of premalignant precursors lesions and also detection of CRC in the early stages of disease, which will impact the treatment outcome. Additionally, screening is expected to impact on the incidence and also shift the distribution of stages of diseases at diagnosis. To tackle the issue of increasing incidence of CRC, the Ministry of Health, Brunei Darussalam is in the process of implementing a CRC screening program.

In conclusion, the study showed that survival rates of CRC patients in Brunei Darussalam were comparable with those of some Asian countries. The present study suggests that CRC patients of older age (≥70), regional stage, distant metastasis stage, Malay ethnicity, other histological type should be considered at high-risk for short survival. Health authorities should implement effective health campaigns in order to raise awareness on CRC and health education programs emphasizing the importance of screening and early diagnosis thus lowering both the incidence and mortality rates due to CRC in Brunei Darussalam.

## References

[B1] Al-Ahwal MS, Shafik YH, Al-Ahwal HM (2013). First national survival data for colorectal cancer among Saudis between 1994 and 2004: what’s next?. BMS Public Health.

[B2] Arnold M, Sierra MS, Laversanne M (2017). Global patterns and trends in colorectal cancer incidence and mortality. Gut.

[B3] Center MM, Jemal A, Smith RA, Ward E (2009). Worldwide variations in colorectal cancer. CA Cancer J Clin.

[B4] Chiu B, Ji BT, Dai Q (2003). Dietary factors and risk of colon cancer in Shanghai, China. Cancer Epidemiol Biomarkers Prev.

[B5] Chou CL, Chang SC, Lin TC (2011). Differences in clinicopathological characteristics of colorectal cancer in younger age and elderly patients: an analysis of 322 patients from a single institution. Am J Surg.

[B7] Du WB, Chia KS, Sankaranarayanan R (2002). Population-based survival analysis of colorectal cancer patients in Singapore, 1968-1992. Int J Cancer.

[B9] Ghazali AK, Musa KI, Naing NN (2010). Prognostic factors in patients with colorectal cancer at Hospital Universiti Sains Malaysia. Asian J Surg.

[B10] Giovannucci E, Willett WC (1994). Dietary factors and risk of colon cancer. Ann Med.

[B11] Goss PE, Strasser-Weippl K, Lee-Bychkovsky BL (2014). Challenges to effective cancer control in China, India, and Russia. Lancet Oncol Commission.

[B12] Harrison LE, Guillem JG, Paty P, Cohen AM (1997). Preoperative carcinoembyronic antigen predicts outcomes in node-negative colon cancer patients: a multivariate analysis of 572 patients. J Am Coll Surg.

[B13] Hasan MR, Suan MA, Soelar SA (2016). Survival analysis and prognostic factors for colorectal cancer patients in Malaysia. Asian Pac J Cancer Prev.

[B14] Ji BT, Deresa SS, Chow WH, Jin F, Gao YT (1998). Colorectal cancer incidence trends by subsite in urban Shanghai, 1972-1994. Cancer Epidemiol Biomarkers Prev.

[B15] Koh KS, Telisinghe PU, Bickle I (2015). Characteristics of young colorectal cancer in Brunei Darussalam: an epidemiologic study of 29 years (1986-2014). Asian Pac J Cancer Prev.

[B16] Kong CK, Roslani AC, Law CW, Law SC, Arumugam K (2010). Impact of socioeconomic on colorectal cancer patients outcomes in Kuala Lumpur and Kuching, Malaysia. Asian Pac J Cancer Prev.

[B17] Laohavinij S, Maneechavakajorn J, Techatanol P (2010). Prognostic factors for survival in colorectal cancer patients. J Med Assoc Thai.

[B18] Lin J, Qiu M, Xu R, Dobs AS (2015). Comparison of survival and clinicopathological features in colorectal cancer among African-Americans, Caucasians and Chinese patients treated in the United States: results from the surveillance epidemiology and end results (SEER) database. Oncotarget.

[B19] Magaji BA, Foong MM, Roslani AC, Law CW (2017). Survival rate and predictors of survival among colorectal cancer patients in a Malaysia tertiary hospital. BMC.

[B20] Mehrkhani F, Nasiri S, Donboli K (2009). Prognostic factors in survival of colorectal cancer patients after surgery. Colorectal Dis.

[B21] Mekenkamp LJ, Heesterbeek KJ, Koopman M (2012). Mucinous adenocarcinomas: poor prognosis in metastatic colorectal cancer. Eur J Cancer.

[B23] O’Connell JB, Maggard MA, Livingston EH, Yo CK (2004). Colorectal cancer in the young. Am J Surg.

[B24] Ong SK, Alikhan F, Lai DTC (2018). Population based lifetime risk estimation of malignant cancers in Brunei Darussalam. Brunei Int Med J.

[B25] Park IJ, Choi GS, Lim KH (2009). Serum carcinoembryonic antigen monitoring after curative resection for colorectal cancer: clinical significance of the preoperative level. Ann Surg Oncol.

[B26] Phimha S, Promthet S, Suwanrungruang K (2019). Health Insurance and colorectal cancer Survival in Khon Kaen, Thailand. Asian Pac J Cancer Prev.

[B27] Sepulveda AR, Hamilton SR, Allegra CJ (2017). Molecular Biomarkers for the Evaluation of colorectal cancer: Guideline from the American Society for Clinical Pathology, College of American Pathologists, Association for Molecular Pathology, and the American Society of Clinical Oncology. J Clin Oncol.

[B28] Sharkas GF, Arquob K, Khader YS (2017). Colorectal cancer in Jordan: Survival Rate and Its Related Factor. J Oncol.

[B29] Shin A, Jung KW, Won YJ (2013). Colorectal cancer mortality in Hong Kong of China, Japan, South Korea and Singapore. World J Gastroenterol.

[B30] Thirunavukarasu P, Sukumar S, Sathaiah M (2011). C-stage in colon cancer: implications of carcinoembryonic antigen biomarker in staging, prognosis, and management. J Natl Cancer Inst.

[B31] Thirunavukarasu P, Talati C, Munial S (2015). Effect of incorporation of pretreatment serum carcinoembryonic antigen levels into AJCC staging for colon cancer on 5-year survival. JAMA Surg.

[B32] Toyoda Y, Nakayama T, Ito Y (2009). Trends in colorectal cancer incidence by subsite in Osaka, Japan. Jpn J Clin Oncol.

[B33] Veettil SK, Lim KG, Chaiyakunapruk N (2016). Colorectal cancer in Malaysia: Its burden and implications for a multiethnic country. Asian J Surg.

[B36] Yuan Y, Li MD, Hu HG (2013). Prognostic and survival analysis of 837 Chinese colorectal cancer. World J Gastroenterol.

[B37] Zhang M, Zhao QC, Liu YP (2014). Prognostic analysis and comparison of colon cancer in Han and Hui patients. World J Gastroenterol.

[B38] Zhang S, Gao F, Luo J, Yang J (2010). Prognostic factors in survival of colorectal cancer patients with synchronous liver metastasis. Colorectal Dis.

